# Global Scenarios of Air Pollutant Emissions from Road Transport through to 2050

**DOI:** 10.3390/ijerph8073032

**Published:** 2011-07-22

**Authors:** Takayuki Takeshita

**Affiliations:** Transdisciplinary Initiative for Global Sustainability, The University of Tokyo, 7-3-1 Hongo, Bunkyo-ku, Tokyo 113-8654, Japan; E-Mails: takeshita@ir3s.u-tokyo.ac.jp or takeshita.t.h@gmail.com; Tel.: +81-3-5841-8576; Fax: +81-3-5841-1545

**Keywords:** air pollutants, road transport, global energy system model

## Abstract

This paper presents global scenarios of sulphur dioxide (SO_2_), nitrogen oxides (NO_x_), and particulate matter (PM) emissions from road transport through to 2050, taking into account the potential impacts of: (1) the timing of air pollutant emission regulation implementation in developing countries; (2) global CO_2_ mitigation policy implementation; and (3) vehicle cost assumptions, on study results. This is done by using a global energy system model treating the transport sector in detail. The major conclusions are the following. First, as long as non-developed countries adopt the same vehicle emission standards as in developed countries within a 30-year lag, global emissions of SO_2_, NO_x_, and PM from road vehicles decrease substantially over time. Second, light-duty vehicles and heavy-duty trucks make a large and increasing contribution to future global emissions of SO_2_, NO_x_, and PM from road vehicles. Third, the timing of air pollutant emission regulation implementation in developing countries has a large impact on future global emissions of SO_2_, NO_x_, and PM from road vehicles, whereas there is a possibility that global CO_2_ mitigation policy implementation has a comparatively small impact on them.

## 1. Introduction

Global climate change has been receiving increasing attention lately. However, reducing emissions of air pollutants [such as sulphur dioxide (SO_2_), nitrogen oxides (NO_x_), and particulate matter (PM)] is a more urgent problem, particularly for developing countries. Of all the emitting sectors, the road transport sector is one of the principal emitters of air pollutants. For example, road transport accounts for 22.5% and 21.2% of total global NO_x_ and PM emissions in 2000, respectively [1–3]. More importantly, road vehicle exhaust emissions are concentrated in urban areas and thus have large health impacts [4,5]. Therefore, national and international efforts to reduce air pollutant emissions from road transport should be given high priority to mitigate air pollution and resulting public health damages.

Projecting possible future global scenarios of road transport’s air pollutant emissions provides important information for the design of related regulatory and technology policies. However, only a little publicly available literature has carried out the projections of air pollutant emissions from road transport on a global scale [1,6,7]. There are mainly two shortcomings in these previous studies. First, the projections of SO_2_ emissions from road transport are given only by [6]. Second, the impacts of climate mitigation policies on air pollutant emissions from road transport are not evaluated.

In this context, the objective of this study is to provide several global scenarios of SO_2_, NO_x_, and PM emissions from road transport through to 2050, taking into account the potential impacts of (1) the timing of air pollutant emission regulation implementation in developing countries; (2) global CO_2_ mitigation policy implementation; and (3) vehicle cost assumptions, on study results. To achieve this objective, the global energy system model REDGEM70, which treats the transport sector in detail, is used.

## 2. Methodology

### 2.1. Overview of the REDGEM70 Model

REDGEM70 (which is an acronym for a Regionally Disaggregated Global Energy Model with 70 regions) is a bottom-up, dynamic linear programming model with a detailed technological representation. The model tailored to this application is designed to determine the cost-optimal energy strategy (e.g., the cost-optimal choice of energy technologies) for each of 70 world regions at 10-year intervals from 2000 to 2050. The objective function is to minimize total discounted energy system costs under the constraints of the satisfaction of exogenously given energy end-use demands, the availability of primary energy resources, the maximum allowable market penetration rate of new technologies, etc. For a more detailed description of REDGEM70, see [8–10].

**Figure 1:**
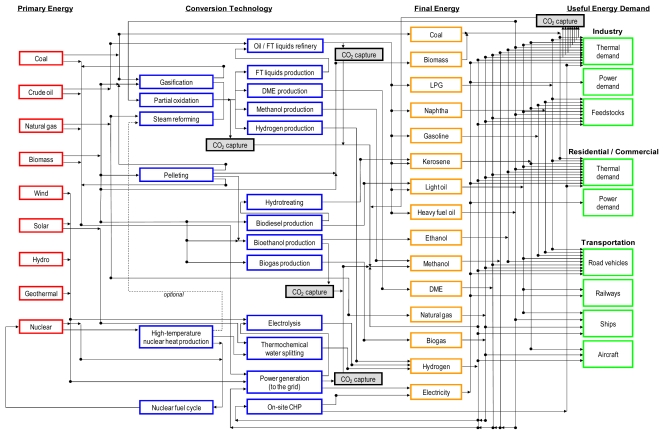
Schematic representation of the structure of REDGEM70. FT: Fischer Tropsch; DME: dimethyl ether; LPG: liquefied petroleum gas; CHP: combined heat and power.

Figure 1 schematically illustrates the structure of REDGEM70. Future trajectories for energy end-use demands were estimated as a function of those for socio-economic driving forces such as population and income in the “Middle Course” case B developed by [11]. For each energy end-use demand category, the possibility of price-induced demand reductions, substitutability among final energy carriers, and changes in efficiency and costs associated with final energy substitution are considered in the model. On the other hand, assumptions on the availability and extraction cost of fossil fuel resource bases were derived from the estimates by [12]. Given aggregate quantity-cost curves for global fossil fuel resource bases, technology and fuel choices and shadow prices of energy carriers are determined simultaneously and endogenously for each model region in the model.

**Figure 2:**
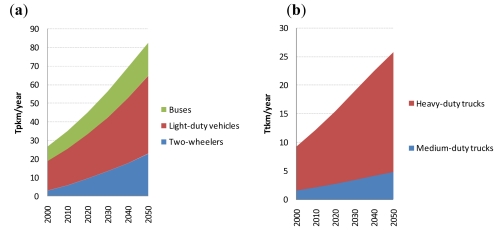
Projected global passenger (a) and freight (b) road transport demand.

REDGEM70 considers the entire supply chain of final energy carriers, which includes primary energy production, interregional energy transportation, energy storage, conversion into secondary energy, intraregional secondary energy distribution, and final energy supply at retail sites (e.g., refueling). The model comprehensively takes into account alternative transport fuels such as conventional biofuels (*i.e.*, bioethanol, biodiesel, and biogas), synthetic fuels (*i.e*., hydrogen, DME, and FT synfuels), natural gas, and electricity, and also technologies for producing them. The model considers the capital and operating and maintenance (O&M) costs separately at each stage of the final energy supply chain by treating the corresponding infrastructure explicitly. The refinery process streams for crude oil and raw FT liquids are described in detail in the model, in which hydro-desulphurization processes of different oil fractions are included. Furthermore, the model considers the difference in the cost of local secondary energy distribution not only by energy carrier, but also by time period, region, and energy end-use sector. This is done by taking into account the difference in the density of final energy demands by energy end-use sector. These features help the model better represent the economics of transport fuels. Table 1 shows the intraregional distribution and refueling costs for each fuel for road transport (for the classification of transport modes, see Figure 2 below). A detailed description of the data and assumptions for the other stages of the final energy supply chain is given in [8–10].

### 2.2. Overview of the Transport Sector Submodel

As regards energy end-use demands in the transport sector, future transport activity demands (in passenger-km (pkm) per year or tonne-km (tkm) per year) were projected for each of 12 transport modes (see Figure 2), which are exogenously given to the model. The demand for two-wheeler travel was projected using the model of [1]; other passenger transport activity demand was projected using the Schafer and Victor model [24]; and freight transport activity demand was projected using the models of [1,13,25]. As mentioned above, future trajectories for population and income in the case B [11] were used as an input to these models for projecting future transport activity demands. Modal shift is fully taken into account using the models of [13,24,25] (the results of their models predicting that people shift to faster transport modes as their total mobility rises). The road traffic supply-demand constraints are given by Equation 1, which determines the number of road vehicles required to meet transport activity demand.

All transport technologies, which refer to possible combinations of propulsion systems and transport fuels in this paper, are characterized by parameters such as on-road fuel economy (in MJ/vehicle-km), capital cost, and O&M cost, and their cost-optimal mix is endogenously determined for each transport mode in the model. The capital vintage structure of 12 transport modes is represented in the model by assuming that the average lifetime is 10 years for motorized two-wheelers and light-duty vehicles [26–28] and 15 years for buses and trucks. However, differences exist in the data for the average lifetime of road vehicles between previous studies. Hence, detailed investigation of these data and incorporating them into the model are one of the future issues.

(1) $$Ract(m,i,t)-S(m,i,t)\leq \sum_{s}\sum_{v}LF(m,i,t)*ADT(m,i,t)*vin(m,s,t)*V(m,v,i,s)$$

where *Ract*(*m*,*i*,*t*) is the demand for road transport (in pkm/year or tkm/year) carried by mode *m* in region *i* at time period *t; S*(*m*,*i*,*t*) is the price-induced transport activity demand reductions in mode *m* in region *i* at time period *t; LF*(*m*,*i*,*t*) is the vehicle occupancy rate for mode *m* in region *i* at time period *t; ADT*(*m*,*i*,*t*) is the annual distance traveled per vehicle for mode *m* in region *i* at time period *t; vin*(*m*,*s*,*t*) is the remaining rate of transport technologies of vintage *s* available for mode *m* in their fleet stocks at time period *t*; and *V*(*m*,*ν*,*i*,*s*) is the number of transport technologies *ν* available for mode *m* in region *i* produced at time period *s*. The values for *LF*(*m*,*i*,*t*) and *ADT*(*m*,*i*,*t*) were estimated and projected for 12 world regions. These values were set to be identical across all model regions belonging to the same world region.

Energy requirements in the transport sector are derived from transport activity (in pkm/year or tkm/year) and actual in-use energy intensity (in MJ/pkm or MJ/tkm) in the model. The actual in-use energy intensities of road vehicles are calculated by dividing their respective on-road fuel economy by their respective average occupancy rate (in passenger/vehicle or tonne/vehicle). It is generally recognized that SO_2_ emissions are a function of the fuel sulphur contents, so SO_2_ emissions from road vehicles are given by Equation 2:

(2) $$Sem(m,i,t)=\sum_{s}\sum_{v}Sef(m,v,i,s)*FE(m,v,i,s)*ADT(m,i,t)*vin(m,s,t)*V(m,v,i,s)$$

where *Sem*(*m*,*i*,*t*) is the SO_2_ emissions from mode *m* in region *i* at time period *t; Sef*(*m*,*ν*,*i*,*s*) is the SO_2_ emission factor for transport technology *ν* available for mode *m* in region *i* produced at time period *s* (in gram/MJ); and *FE*(*m*,*ν*,*i*,*s*) is the on-road fuel economy of transport technology *ν* available for mode *m* in region *i* produced at time period *s*. The values for *Sef*(*m*,*ν*,*i*,*s*) and *FE*(*m*,*ν*,*i*,*s*) were estimated and projected for 12 world regions. These values were set to be identical across all model regions belonging to the same world region.

On the other hand, NO_x_ and PM emissions from road vehicles depend on operating methodology and exhaust emission control technology, and thus are given by Equation 3:

(3) $$NSem(m,p,i,t)=\sum_{s}\sum_{v}NSef(m,p,v,i,s)*ADT(m,i,t)*vin(m,s,t)*V(m,v,i,s)$$

where *NSem*(*m*,*p*,*i*,*t*) is the emissions of pollutant *p* from mode *m* in region *i* at time period *t* and *NSef*(*m*,*p*,*ν*,*i*,*s*) is the emission factor for pollutant *p* for transport technology *ν* available for mode *m* in region *i* produced at time period *s* (in gram/vehicle-km). The values for *NSef*(*m*,*p*,*ν*,*i*,*s*) were estimated and projected for 12 world regions. These values were set to be identical across all model regions belonging to the same world region.

### 2.3. Data and Assumptions for Transport Technologies

**Figure 3:**
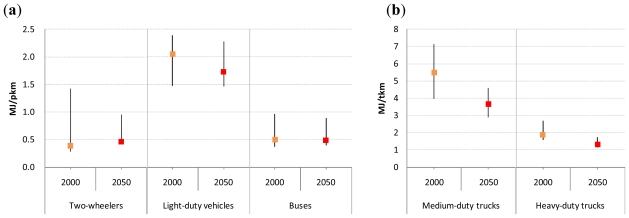
Projected actual in-use energy intensities of baseline passenger (a) and freight (b) road transport technologies. Note: The world averages shown as squares in these figures are calculated as the activity-weighted averages of the actual in-use energy intensity of each road transport technology. The range denotes the difference by region.

The projections of the on-road average fuel economies of baseline road transport technologies (*i.e*., gasoline internal combustion engine (ICE) two-wheelers, gasoline ICE light-duty vehicles, diesel ICE buses, diesel ICE medium-duty trucks, and diesel ICE heavy-duty trucks) by world regions are taken from [1]. Figure 3 shows the actual in-use energy intensities of these baseline road transport technologies for the years 2000 and 2050. Note that the actual in-use energy intensities of the transport technologies produced in 2000 and 2050 are shown in these figures. The actual in-use energy intensities of baseline passenger road transport technologies were estimated to remain roughly constant over the period 2000–2050 because their improved fuel efficiencies would be offset by declining vehicle occupancy rates [29].

By conducting a comprehensive survey of literature and interviewing experts, possible combinations of propulsion systems and transport fuels were defined for each transport mode and model input parameters were set for each transport technology. The assumed possible combinations of propulsion systems and transport fuels (*i.e*., transport technologies) for light-duty vehicles and heavy-duty trucks are listed in Tables 2 and 3, respectively. Technical, economic, and environmental parameters for transport technologies available for the two transport modes are also shown in these tables because they have a great impact on the simulation results. Transport technologies available for medium-duty trucks are assumed to be the same as those available for light-duty vehicles (except for LH_2_ ICEV being available instead of CGH_2_ ICEV), whereas all transport technologies considered for the light-duty vehicle sector except plug-in hybrids are assumed to be available for the bus sector. Alternative transport technologies available for two-wheelers include ICEs powered with gasohol or ethanol and electric vehicles.

There are four important assumptions underlying Table 2. First, pure electric light-duty vehicles are assumed to have a driving range of 200 km, although all other transport technologies available for light-duty vehicles are assumed to have a driving range of 500 km. To compensate for such reduced driving range, pure electric vehicles are assumed to require fast charging stations in cities and along certain corridors [31], which were estimated to add US$_2000_ 6.1/GJ to the delivered cost of electricity (see Table 1) following the method of [19]. Second, plug-in hybrids are assumed to operate as electric vehicles for 65% of their daily driving [30]. Third, the specific cost of Li-ion batteries was estimated to eventually drop to US$_2000_ 407/kWh for conventional hybrids, US$_2000_ 372/kWh for plug-in hybrids, and US$_2000_ 292/kWh for pure electric vehicles with a 200 km range, respectively [31]. Fourth, the specific cost of a proton exchange membrane (PEM) fuel cell stack was estimated to eventually reach US$_2000_ 95/kW [22,30]. For hydrogen storage, the specific cost of a CGH_2_ storage tank at a pressure of 700 bar was estimated to eventually reach US$_2000_ 277/kg [30,32], and that of a LH_2_ storage tank is assumed to eventually reach the same value [40].

On the other hand, there are two important assumptions underlying Table 3. First, a hybrid propulsion system is not considered for heavy-duty trucks because they operate primarily on highways at near to maximum rated power and because hybrids are estimated to provide virtually no efficiency benefits on highway driving cycles [1]. Second, a fuel cell propulsion system, for which durability is a key issue, is not considered for heavy-duty trucks as well because they often travel over 100,000 km/year [1,22].

**Figure 4:**
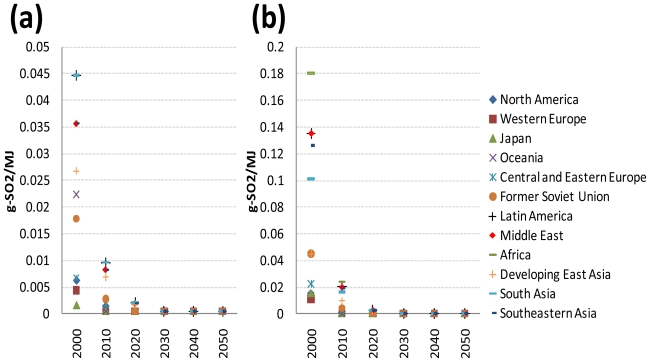
Projected SO_2_ emission factors for gasoline (a) and diesel (b) by world region.

The values for SO_2_ emission factors for gasoline and diesel in 2000 are taken from [3]. Their values were projected for each developed region from information on current and future regulations on the fuel sulphur contents (e.g., the EPA Tier 2 emission standards, the EURO 5 and 6 emission standards, and Japan’s Air Pollution Control Law). Their values for each of the reforming and developing regions were projected based on two assumptions (the regional classification in this study being identical to that of [3,24]). First, the three Asian developing regions will adopt the same vehicle emission standards as in Japan with a 20-year lag [1,24]. Second, the reforming regions and the other developing regions will adopt the same vehicle emission standards as in Western Europe with a lag of 10 and 20 years, respectively [1,24]. Specifically, it is assumed that gasoline and diesel fuels eventually have a maximum sulphur content of 10 ppm (weight basis) in 2010 for the developed regions, in 2020 for the reforming regions, and in 2030 for the developing regions. The SO_2_ emission factor values for the intervening time periods were interpolated assuming a constant reduction rate. In other words, the average annual change rate from 2000 to the time period when the sulphur content of gasoline and diesel fuels reaches 10 ppm was applied to the SO_2_ emission factor values for the intervening time periods. Figure 4a,b shows the resulting projection of SO_2_ emission factors for gasoline and diesel, respectively, by world region. The SO_2_ emission factor ratios of LPG and CNG to petroleum gasoline are 0.19 and 0.03, respectively. For the other road transport fuels the SO_2_ emission factor is assumed to be zero.

**Figure 5:**
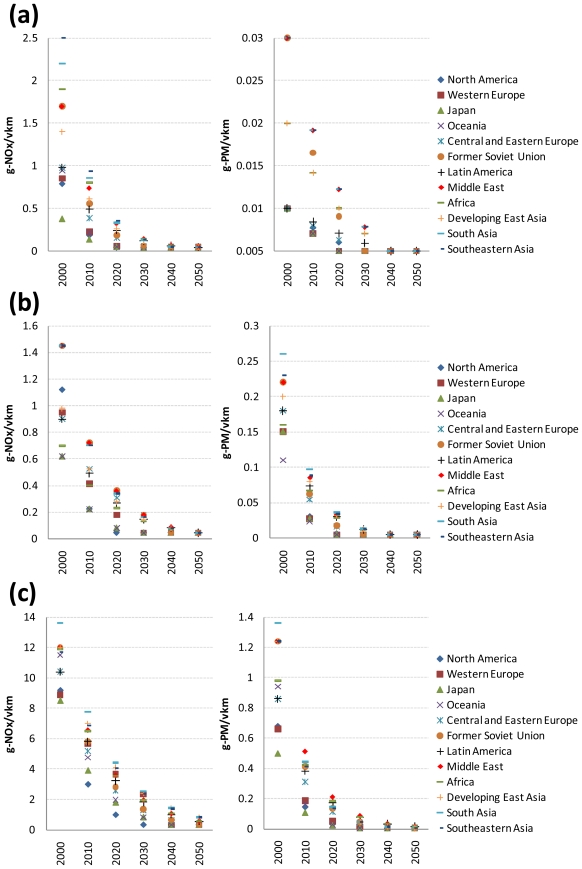
Projected NO_x_ and PM emission factors for gasoline ICE light-duty vehicles (a), diesel ICE light-duty vehicles (b), and diesel ICE heavy-duty trucks (c) by world region.

On the other hand, the values for NO_x_ and PM emission factors for gasoline ICE two-wheelers, gasoline and diesel ICE light-duty vehicles, gasoline and diesel ICE buses, gasoline and diesel ICE medium-duty trucks, and diesel ICE heavy-duty trucks in 2000 are taken from [1,3]. Their values were projected for each developed region from information on current and future related regulations (e.g., the US Federal emissions standards, the EURO 5 and 6 emission standards, and Japan’s post new long-term emission regulations). Their values for each of the reforming and developing regions were projected based on the same two assumptions used for projecting the SO_2_ emission factors. Specifically, it is assumed that NO_x_ and PM emission factors for the developed regions reach the minimum values in specific time periods (in 2040 at the latest). The NO_x_ and PM emission factor values for the intervening time periods were interpolated assuming a constant reduction rate. As an example, Figure 5a–c shows the resulting projection of NO_x_ and PM emission factors for gasoline and diesel ICE light-duty vehicles and diesel ICE heavy-duty trucks, respectively, by world region.

### 2.4. Definition of Simulation Scenarios

In order to observe the effects of: (1) the timing of air pollutant emission regulation implementation in developing countries; (2) global CO_2_ mitigation policy implementation; and (3) vehicle cost assumptions, on the simulation results, five scenarios were set up for each simulation: the Business-as-Usual (BaU) scenario, the CO_2_ mitigation scenario, the BaU scenario with a 10- or 30-year lag, and the CO_2_ mitigation scenario with optimistic cost assumptions. In the BaU scenario, no CO_2_ mitigation policies are assumed to be implemented, whereas the CO_2_ mitigation scenario is constrained to reduce total global CO_2_ emissions by 50% from the 2005 level in 2050 (this constraint being the same as that given in the International Energy Agency’s BLUE scenarios [22]). The CO_2_ mitigation scenario assumes full flexibility in where CO_2_ emissions reduction is achieved to meet the constraint.

On the other hand, as described above, it is assumed, for reference, that the SO_2_, NO_x_, and PM emission factors in the developing regions will reach their values for Japan or Western Europe with a 20-year lag. This time lag was varied at 10 and 30 years for sensitivity purposes. Similar to the reference projections, the SO_2_, NO_x_, and PM emission factor values for the intervening time periods were interpolated assuming a constant reduction rate. In the CO_2_ mitigation scenario with optimistic cost assumptions, the specific costs of the components of hybrid vehicles, electric vehicles, and fuel cell vehicles not included in baseline gasoline or diesel ICEVs (*i.e*., tank for alternative fuels, fuel reformer, hybrid transmission, 1-spd transmission, electric motor and controller, Li-ion batteries, and PEM fuel cell stack) are assumed to reach levels that are 50% lower than their reference specific costs. This scenario corresponds to the situation where technological advancements would be accelerated as a result of global CO_2_ mitigation policy implementation.

## 3. Results and Discussion

### 3.1. Cost-Optimal Choice of Transport Technologies in the Road Transport Sector

**Figure 6:**
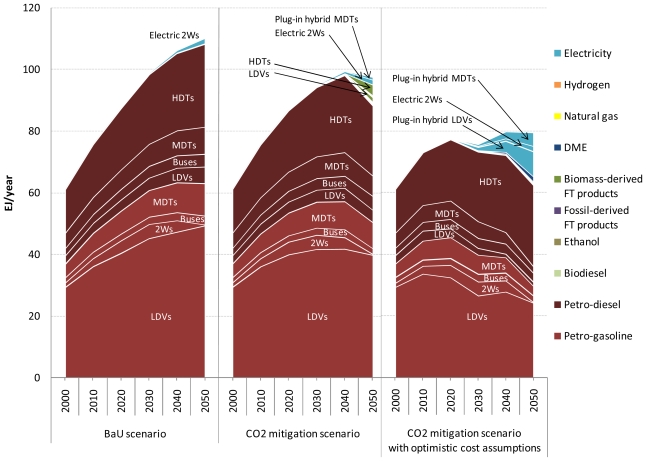
Cost-optimal mix of road transport fuels in the three scenarios. LDVs: light-duty vehicles; 2Ws: two-wheelers; MDTs: medium-duty trucks; HDTs: heavy-duty trucks.

Before discussing the results for air pollutant emissions from road transport, attention is focused on the cost-optimal choice of transport technologies in the five road transport sectors in the BaU and CO_2_ mitigation scenarios with and without the optimistic vehicle cost assumptions. Figure 6 shows the cost-optimal mix of road transport fuels for the three scenarios at the global level. Also, the shadow prices of petroleum gasoline and diesel fuel are shown for the three scenarios in  Appendices.

In the BaU scenario, petroleum products dominate the fuel mix for road transport. However, although not shown here, conventional hybrids are introduced into the light-duty vehicle, bus, and medium-duty truck markets even in this scenario. For example, the share of conventional hybrids in the global light-duty vehicle fleet in this scenario increases from 8.6% in 2020 to 48.5% in 2050 (see Appendices). This result arises because of the increasing price of oil and because of the decreasing purchase price of advanced vehicles. In the three scenarios over the period 2000–2050, global road fuel consumption is highest in the light-duty vehicle sector followed by the heavy-duty truck sector and then by the medium-duty truck sector.

Despite the stringent CO_2_ emissions reduction target used in the CO_2_ mitigation scenario, it should be emphasized that the differences in the results between the BaU and CO_2_ mitigation scenarios are comparatively small. The first reason is that the assumptions about the costs of advanced vehicles are conservative. The second reason is that the marginal CO_2_ abatement cost has been estimated to be higher in the road transport sector than in the other CO_2_-emitting sectors [22,51]. Reflecting this, road transport accounts for only 7.1% of the total global CO_2_ emissions reduction in 2050 in the CO_2_ mitigation scenario compared to the BaU scenario. In this scenario, the majority of the reduction comes from power generation, fuel production, and industrial processes (including petroleum/FT refinery), which is achieved by efficiency improvements, energy and feedstock substitution, and CO_2_ capture and storage. These results are in agreement with the marginal CO_2_ emission abatement cost curve presented by [22].

The choice of road transport technologies in the CO_2_ mitigation scenario is different from that in the BaU scenario in the following respects. First, hybrids penetrate the light-duty vehicle, bus, and medium-duty truck markets more deeply in the CO_2_ mitigation scenario than in the BaU scenario, which results in a lower petroleum fuel consumption by these sectors in the former scenario. In the CO_2_ mitigation scenario, not only conventional hybrids but also plug-in hybrids are introduced into the light-duty vehicle and medium-duty truck markets. Second, biofuels, more specifically biomass-derived FT gasoline and diesel, make a noticeable contribution to total global road fuel consumption, mainly in the light-duty vehicle and heavy-duty truck sectors, in 2050 in the CO_2_ mitigation scenario. In this scenario, CO_2_ emissions from heavy-duty trucks are reduced by replacing petroleum diesel fuel with biodiesel and biomass-derived FT diesel. The first reason for these results is that the shadow prices of petroleum gasoline and diesel fuel become higher in the CO_2_ mitigation scenario than in the BaU scenario. Therefore, energy-efficient transport technologies and low-carbon transport fuels are preferred in the CO_2_ mitigation scenario. The second reason is that as the mandated CO_2_ emissions reduction target becomes more stringent, the carbon price increases to the level that is sufficient to economically justify the deployment of clean but costly technology and fuel options.

In the CO_2_ mitigation scenario with optimistic cost assumptions, the share of hybrids, especially plug-in hybrids, in the global road vehicle fleet increases even further, which reaches almost 100% of the global fleet of light-duty vehicles (100%), buses (97.8%), and medium-duty trucks (99.8%) in 2050. As a consequence, global road fuel consumption in 2050 decreases by 27.8% in this scenario compared to the BaU scenario, and road transport accounts for 15.8% of the total global CO_2_ emissions reduction in 2050 in this scenario compared to the BaU scenario. This implies that lowering the specific cost of Li-ion batteries is important for the penetration of plug-in hybrids. It is interesting to note that the increasing penetration of hybrids leads to the reduction in the number of road vehicles fueled by biomass-derived FT synfuels under the CO_2_ emission constraints. These results indicate that except for electric two-wheelers, pure electric vehicles and fuel cell vehicles are transport technologies that might play a role in the second half of the century and/or in a more carbon-constrained world.

For comparison purposes, the results of sensitivity analysis with respect to the prices of petroleum gasoline and diesel fuel are presented in Appendices. Vehicle costs and delivered costs of road transport fuels are key drivers of the results. As long as fossil fuel prices remain low, petroleum products account for the dominant share of global road fuel consumption.

### 3.2. Air Pollutant Emissions from Road Transport

**Figure 7:**
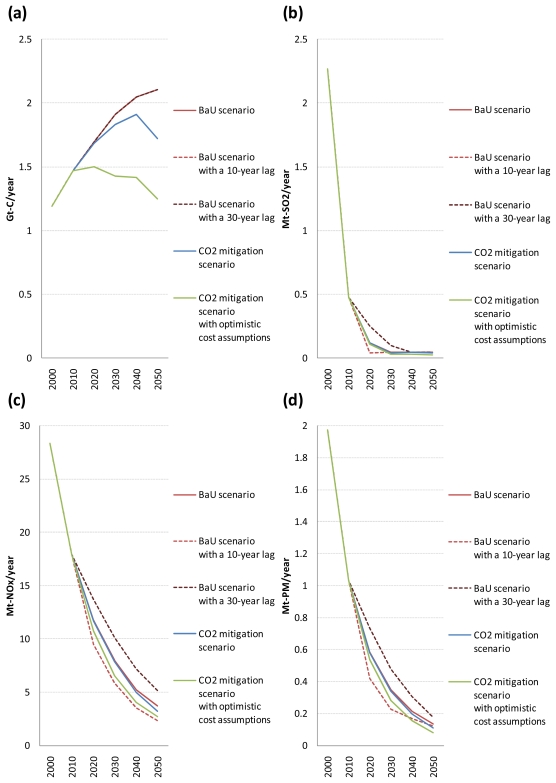
Global emissions of CO_2_ (a), SO_2_ (b), NO_x_ (c), and PM (d) from road vehicles in the five scenarios.

Figure 7a–d shows the global emissions of CO_2_, SO_2_, NO_x_, and PM from road vehicles, respectively, for the five scenarios. Also, the ratios of global emissions of CO_2_, SO_2_, NO_x_, and PM from road vehicles in the four alternative scenarios to those in the BaU scenario are shown only for the period 2020–2050 in Table 4 (because the differences in the results occur between the scenarios from 2020).

As shown in Figure 7, even without CO_2_ emission constraints, global emissions of SO_2_, NO_x_, and PM from road vehicles decrease substantially over time due to an assumed autonomous decline in their emission factors. In the BaU scenario, global emissions of SO_2_, NO_x_, and PM from road vehicles decrease by 97.9%, 87.0%, and 93.1%, respectively, from 2000 to 2050. In particular, the reduction in global road vehicle SO_2_ emissions is very rapid because they depend on the fuel sulphur contents and are not affected by slow vehicle stock turnover. By comparison, the reduction in global road vehicle NO_x_ and PM emissions is gradual. These results are in line with the fact that the fuel sulphur contents, and hence road vehicle SO_2_ emissions, need to be reduced to a very low level for road vehicle NO_x_ and PM emissions to be reduced sufficiently because sulphur poisons catalysts for automotive exhaust emission control systems.

It can be seen from Figure 7 that the timing of air pollutant emission regulation implementation in the developing regions has an evident impact on future trajectories for global SO_2_, NO_x_, and PM emissions from road vehicles. Cumulative global emissions of SO_2_, NO_x_, and PM from road vehicles over the period 2010–2050 increase by 25.3%, 16.2%, and 18.0%, respectively, if the developing regions adopt the same vehicle emission standards as in the developed regions 10 years later than the BaU scenario, and decrease by 11.0%, 16.0%, and 14.8%, respectively, if the developing regions adopt them 10 years earlier than that scenario. Of all time periods, the largest difference between the BaU scenarios with a 10- and 30-year lag is by a factor of 6.4 (in 2020) for global road vehicle SO_2_ emissions, 2.2 (in 2050) for global road vehicle NO_x_ emissions, and 2.1 (in 2030) for global road vehicle PM emissions. These results suggest that an early implementation of sufficiently stringent regulations on road vehicle exhaust emissions could have a noticeable positive effect on mitigating health damages in the developing regions.

In contrast, Figure 7 and Table 4 show that as long as the costs of advanced vehicles take the reference values, global CO_2_ mitigation policy implementation has a small impact on global emissions of SO_2_, NO_x_, and PM from road vehicles. There are three main reasons for this. First, there is a small difference in the choice of transport technologies in the road transport sector between the BaU and CO_2_ mitigation scenarios. Second, biomass-derived FT synfuels, which are chosen as an alternative fuel for road transport in the CO_2_ mitigation scenario, have similar emission factors for NO_x_ and PM to those of petroleum products (see Tables 2 and 3). This implies that the use of liquid biofuels to reduce CO_2_ emissions does not necessarily lead to the reduction in emissions of air pollutants other than SO_2_. Third, a substantial reduction in global SO_2_, NO_x_, and PM emissions from road vehicles over time, which is caused by an assumed autonomous decline in their emission factors, makes the differences in global road vehicle exhaust emissions between the BaU and CO_2_ mitigation scenarios very small in absolute terms. However, if the costs of advanced vehicles are assumed to take their optimistic values under the CO_2_ emission constraints, the picture changes. The differences in global road vehicle exhaust emissions between the BaU and CO_2_ mitigation scenarios with optimistic cost assumptions become considerable both in absolute and relative terms.

As shown in Table 4, the reduction rate of global road vehicle SO_2_ emissions in the CO_2_ mitigation scenario compared to the BaU scenario is almost the same as that of global road vehicle CO_2_ emissions. This is because the CO_2_ and SO_2_ emission factors for road vehicles powered by biomass-derived FT synfuels or electricity, both of which are chosen as an alternative fuel for road transport in the CO_2_ mitigation scenario, are zero. On the other hand, the reduction rate of global road vehicle NO_x_ and PM emissions is lower than that of global road vehicle CO_2_ and SO_2_ emissions (except for the reduction rate of global road vehicle NO_x_ emissions in 2050). The main reason for this is that the reduction rate of global NO_x_ and PM emissions from heavy-duty trucks is lower than that of global CO_2_ and SO_2_ emissions from heavy-duty trucks because the CO_2_ and SO_2_ emission factors for heavy-duty trucks fueled by biomass-derived FT diesel are zero while the NO_x_ and PM emission factors for them are similar to those for petroleum diesel-fueled heavy-duty trucks. However, the reduction in global road vehicle NO_x_ and PM emissions in the CO_2_ mitigation scenario compared to the BaU scenario is larger than that in global road vehicle SO_2_ emissions in absolute terms because global emissions of SO_2_ from road vehicles are much smaller than those of NO_x_ and PM in the BaU scenario.

### 3.3. Results for Air Pollutant Emissions by Mode

**Figure 8:**
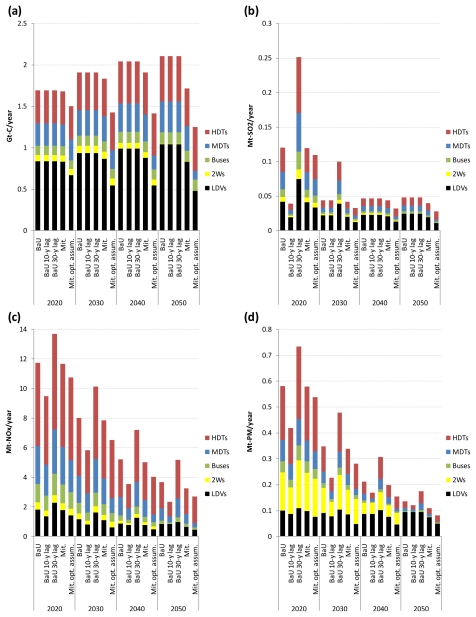
Breakdown of global emissions of CO_2_ (a), SO_2_ (b), NO_x_ (c), and PM (d) from road vehicles by mode in the five scenarios.

For the same reason as in Table 4, the sectorally disaggregated results are shown on a global scale for the five scenarios for the period 2020–2050 in Figure 8a–d. In all the scenarios, the share of light-duty vehicles and heavy-duty trucks in global emissions of CO_2_, SO_2_, NO_x_, and PM from road vehicles is large and increases over time, reflecting their large share of total global road fuel consumption. Their share of global road vehicle exhaust emissions in 2020 ranges from 53.2% to 63.9% in the BaU scenario and from 53.1% to 63.7% in the CO_2_ mitigation scenario depending on the pollutant, whereas their share in 2050 ranges from 71.1% to 87.7% in the BaU scenario and from 73.4% to 88.7% in the CO_2_ mitigation scenario depending on the pollutant. These results indicate that policies should be targeted at the light-duty vehicle and heavy-duty truck sectors to achieve further substantial reduction in global road vehicle exhaust emissions below the 2050 emissions levels presented here.

Attention is then focused on which mode is accounting for the largest share of the increase (reduction) in global road vehicle exhaust emissions caused by delayed (early) implementation of vehicle emission control regulations. The increase in global road vehicle exhaust emissions in the BaU scenario with a 30-year lag compared to the BaU scenario is largest in the heavy-duty truck sector, regardless of time periods and the type of pollutant. In particular, the increase in global NO_x_ and PM emissions from heavy-duty trucks caused by the delay in regulation implementation is large. Similarly, the reduction in global road vehicle exhaust emissions in the BaU scenario with a 10-year lag compared to the BaU scenario is largest in the heavy-duty truck sector, regardless of time periods and the type of pollutant.

In all the scenarios, the following findings can also be obtained from Figure 8. First, the light-duty vehicle sector makes a comparatively large contribution to global road vehicle SO_2_ and PM emissions. Second, the two-wheeler sector is one of the largest PM emitters in the road transport sector. Third, the bus and medium-duty truck sectors make a comparatively large contribution to global road vehicle NO_x_ and SO_2_ emissions. Fourth, the heavy-duty truck sector is the largest NO_x_ emitter in the road transport sector.

## 4. Conclusions

In this paper, global scenarios of SO_2_, NO_x_, and PM emissions from road transport have been presented through to 2050, taking into account the potential impacts of (1) the timing of air pollutant emission regulation implementation in developing countries; (2) global CO_2_ mitigation policy implementation; and (3) vehicle cost assumptions, on study results. This was done by using the global energy system model REDGEM70 treating the transport sector in detail. The major findings and implications obtained can be summarized as follows.

First, as long as the reforming and developing regions adopt the same stringent vehicle emission standards as in the developed regions within a 30-year lag, global emissions of SO_2_, NO_x_, and PM from road vehicles decrease substantially over time. In the BaU scenario (where the reforming and developing regions are assumed to adopt them with a lag of 10 and 20 years, respectively), global emissions of SO_2_, NO_x_, and PM from road vehicles decrease by 97.9%, 87.0%, and 93.1%, respectively, from 2000 to 2050. For this to occur in the real world, investment in reducing the sulphur contents of petroleum products for road transport to or below 10 ppm should be started as soon as possible in all countries. It should also be kept in mind that the assumptions on the timing of air pollutant emission regulation implementation in the developing regions are somewhat optimistic, and that the results change if such regulation is assumed to be implemented much later or not to work well.

Second, as expected, the timing of air pollutant emission regulation implementation in the developing regions has an evident impact on future global emissions of SO_2_, NO_x_, and PM from road vehicles. The adoption of the same vehicle emission standards as in the developed regions by the developing regions 10 years later (earlier) than the BaU scenario increases (decreases) cumulative global emissions of SO_2_, NO_x_, and PM from road vehicles over the period 2010–2050 by 25.3% (11.0%), 16.2% (16.0%), and 18.0% (14.8%), respectively. This means that an early implementation of stringent regulations on road vehicle exhaust emissions could contribute significantly to mitigating health damages in the developing regions.

Third, there is a possibility that global CO_2_ mitigation policy implementation has a comparatively small impact on future global road vehicle exhaust emissions. This is because of the small difference in the choice of road transport technologies between the BaU and CO_2_ mitigation scenarios, which is caused by high marginal CO_2_ abatement costs in the road transport sector. The reduction rate of global road vehicle NO_x_ and PM emissions in the CO_2_ mitigation scenario compared to the BaU scenario is lower than that of global road vehicle SO_2_ emissions. It is important to note that the use of liquid biofuels does not necessarily lead to the reduction in NO_x_ and/or PM emissions.

Fourth, in all the scenarios considered here, light-duty vehicles and heavy-duty trucks make a large and increasing contribution to future global emissions of SO_2_, NO_x_, and PM from road vehicles, reflecting their large share of total global road fuel consumption. The increase (reduction) in future global road vehicle exhaust emissions caused by the developing regions’ 10-year delay (precedence) in the adoption of the same vehicle emission standards as in the developed regions is largest in the heavy-duty truck sector, regardless of time periods and the type of pollutant. Therefore, to achieve further substantial reduction in future global road vehicle exhaust emissions below the levels presented here, effective policies aimed at these sectors, such as promoting R&D for increasing the number of cost-effective alternatives to diesel-fueled heavy-duty trucks, should be put forward.

## Figures and Tables

**Figure A1:**
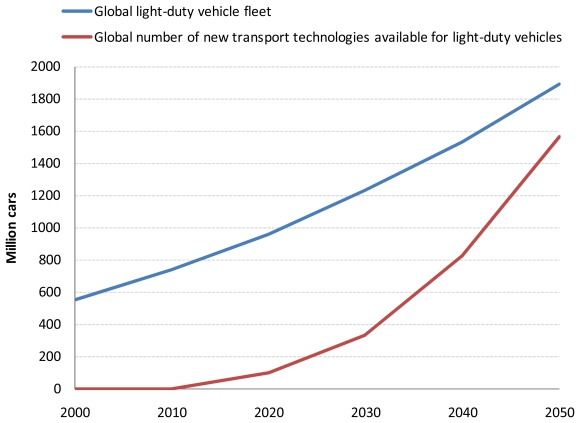
Assumed maximum allowable market penetration of new transport technologies available for light-duty vehicles that are not existent until now.

**Figure A2:**
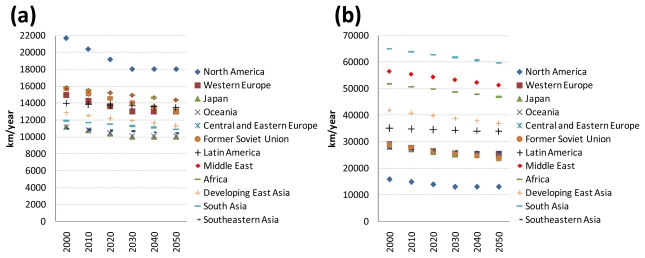
Assumed average annual distance traveled per vehicle for light-duty vehicles (a) and buses (b) by world region.

**Figure A3:**
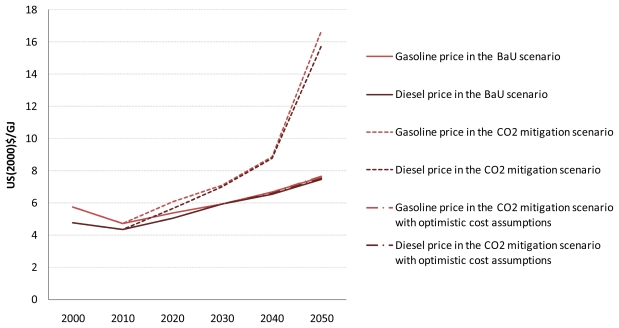
Shadow prices of petroleum gasoline and diesel fuel in the three scenarios.

**Figure A4:**
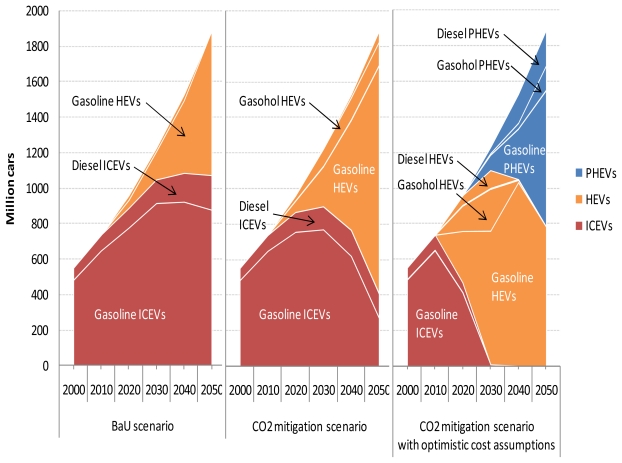
Cost-optimal mix of transport technologies available for light-duty vehicles in the three scenarios.

**Figure A5:**
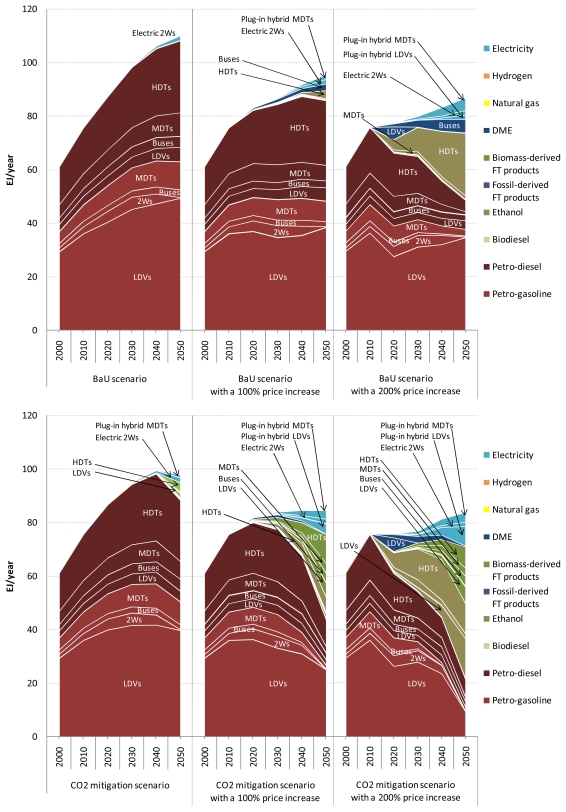
Cost-optimal mix of road transport fuels in the six scenarios.

**Figure A6:**
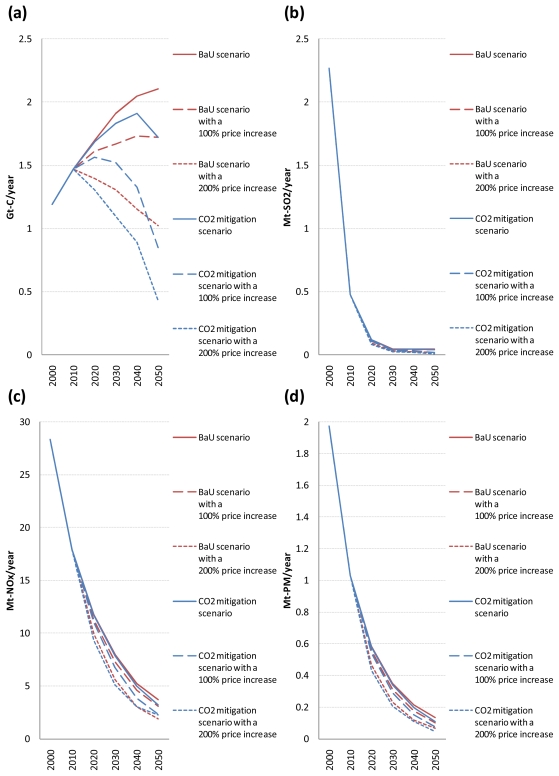
Global emissions of CO_2_ (a), SO_2_ (b), NO_x_ (c), and PM (d) from road vehicles in the six scenarios.

**Table 1:** Intraregional distribution and refueling costs for fuels for road transport 1.

Transport fuel	Intraregional distribution cost 2 (US$_2000_/GJ) 2000/2050	Refueling cost 2 (US$_2000_/GJ)
Gasoline, gasohol 3, diesel (including biodiesel), and FT products	1.0 4	1.6
LPG	1.5 4	2.8
Ethanol	1.3 4	2.3
DME	2.1 4	3.9
Liquid hydrogen (LH_2_)		
LH_2_ delivery and gaseous H_2_ (GH_2_) refueling	3.1 4	6.9
LH_2_ delivery and LH_2_ refueling		
LH_2_ supply to medium-duty trucks	3.1 4	6.1
Compressed natural gas (CNG)		
CNG supply to light-duty vehicles and heavy-duty trucks	2.1–4.8/2.0–2.9 5	4.0
CNG supply to buses and medium-duty trucks	1.3–2.9/1.2–1.7 5,6	4.0
Compressed GH_2_ (CGH_2_)		
Centralized H_2_ production		
CGH_2_ supply to light-duty vehicles	3.0–6.8/2.9–4.1 5	5.8
CGH_2_ supply to buses and medium-duty trucks	1.8–4.1/1.7–2.5 5,6	5.8
Decentralized H_2_ production	–	4.8
Electricity		
Electricity supply to two-wheelers and light-duty vehicles	3.3–7.4/3.1–4.4 5	6.1
Electricity supply to buses and medium-duty trucks	2.0–4.4/1.9–2.6 5,6	6.1

1 Data are taken from [13–23];

2 The share of capital costs in total costs is assumed to be 85% for pipeline distribution of CNG and CGH_2_ and electric power transmission, whereas the corresponding estimate is 33% for truck distribution of liquid fuels and 75% for refueling [15,19];

3 Gasohol is defined as a 10% ethanol to 90% gasoline volumetric blend;

4 Costs of distributing liquid transport fuels by truck are assumed to be the same across all transport modes because the distribution distance has a small impact on them [15,19];

5 The range of these parameter values denotes the difference by region. Following the method of [17], they vary by region and over time as a function of the percentage of population living in urban areas. They are estimated to be lower for urban areas where a geographically concentrated demand exists;

6 Considering that buses and urban delivery trucks are usually centrally refueled, costs of distributing CNG, CGH_2_, and electricity to buses and medium-duty trucks are assumed to be 40% lower than those of distributing them to light-duty vehicles.

**Table 2:** Input parameters for transport technologies available for light-duty vehicles.

Transport technology 1	Vehicle fuel economy ratio 2 2000/2050	Vehicle cost 3 (US$_2000_/vehicle) 2000/2050	NO_x_ emission factor ratio 4		PM emission factor ratio 5	

			Relative to gasoline ICEV	Relative to diesel ICEV	Relative to gasoline ICEV	Relative to diesel ICEV
Gasoline ICEV	1.00	18,000	1.00 6	–	1.00 6	–
Diesel ICEV	0.850	19,560	–	1.00 6,7	–	1.00 6,7
LPG ICEV	0.923	19,750	0.860	–	0.200	–
Gasohol ICEV	0.996	18,000	1.01 6	–	0.936 6	–
Ethanol ICEV	0.949	18,970	1.24	–	0	–
DME ICEV	0.850	20,310	–	0.420	–	0.250
CNG ICEV	0.952	19,780	1.05	–	0.200	–
CGH_2_ ICEV	0.885	26,050/22,450	1.01	–	0	–

Gasoline HEV	0.700/0.568	22,500/18,900	0.500 6	–	0.500 6	–
Diesel HEV	0.640/0.519	24,440/20,530	–	0.500 6,7	–	0.500 6,7
LPG HEV	0.646/0.524	24,690/20,740	0.430	–	0.100	–
Gasohol HEV	0.697/0.568	22,500/18,900	0.505 6	–	0.468 6	–
Ethanol HEV	0.665/0.539	23,720/19,920	0.620	–	0	–
DME HEV	0.640/0.519	25,370/21,310	–	0.210	–	0.125
CNG HEV	0.667/0.541	24,730/20,770	0.525	–	0.100	–
CGH_2_ HEV	0.619/0.503	26,570/23,570	0.505	–	0	–

Gasoline PHEV	0.418/0.435	73,800/22,950	0.350 6	–	0.350 6	–
Diesel PHEV	0.384/0.400	75,740/24,580	–	0.350 6,7	–	0.350 6,7
Gasohol PHEV	0.418/0.434	73,800/22,950	0.354 6	–	0.328 6	–
Ethanol PHEV	0.407/0.423	75,020/23,970	0.434	–	0	–

Gasoline FCHV	0.578/0.516	303,900/33,740	0.200 6	–	0	–
DME FCHV	0.513/0.457	278,300/30,910	0.200	–	0	–
CGH_2_ FCHV	0.381/0.340	246,700/25,690	0	–	0	–

BEV	0.286/0.310	152,600/27,640	0	–	0	–

1 ICEV = internal combustion engine vehicle; HEV = hybrid electric vehicle; PHEV = plug-in hybrid electric vehicle; FCHV = fuel cell hybrid vehicle; BEV = battery electric vehicle;

2 Data are taken from [1,30–39];

3 Data are taken from [22,25,30–32,34–38,40–46]. Vehicle cost values were set to be identical across all model regions because the vehicle market is becoming increasingly global [43] and because of a lack of detailed regional data;

4 Data are taken from [1,33–35,47];

5 Data are taken from [1,33,35];

6 The NO_x_ emission factor for vehicles fueled by FT gasoline/diesel is assumed to be 27% lower than that for vehicles fueled by petroleum gasoline/diesel, while the PM emission factor for vehicles fueled by FT gasoline/diesel is assumed to be 21% lower than that for vehicles fueled by petroleum gasoline/diesel [48];

7 The NO_x_ emission factor for vehicles fueled by biodiesel is assumed to be 10% higher than that for vehicles fueled by petroleum diesel, while the PM emission factor for vehicles fueled by biodiesel is assumed to be 75% lower than that for vehicles fueled by petroleum diesel [35].

**Table 3:** Input parameters for transport technologies available for heavy-duty trucks.

Transport technology	Vehicle fuel economy ratio 1	Vehicle cost 2 (US$_2000_/vehicle)	NO_x_ emission factor ratio 3	PM emission factor ratio 4
Diesel ICEV	1.00	143,000	1.00 5,6	1.00 5,6
Ethanol ICEV	1.03	144,800	0.406	0
DME ICEV	1.00	159,600	0.420	0.250
CNG ICEV	1.13	153,800	0.292	0.006

1 Data are taken from [49,50];

2 Data are taken from [34,40,45,50]. Vehicle cost values were set to be identical across all model regions because the vehicle market is becoming increasingly global [43] and because of a lack of detailed regional data;

3 Data are taken from [3,33,34];

4 Data are taken from [3,33];

5 Same as footnote 6 in Table 2;

6 Same as footnote 7 in Table 2.

**Table 4:** Ratios of global emissions of CO_2_, SO_2_, NO_x_, and PM from road vehicles in the four alternative scenarios to those in the BaU scenario 1

	CO_2_ emissions ratio (%)				SO_2_ emissions ratio (%)				NO_x_ emissions ratio (%)				PM emissions ratio (%)			

	2020	2030	2040	2050	2020	2030	2040	2050	2020	2030	2040	2050	2020	2030	2040	2050
BaU scenario with a 10-year lag																
Light-duty vehicles	100.0	100.0	100.0	100.0	47.2	100.0	100.0	100.0	74.6	68.0	87.6	100.0	88.2	85.8	100.0	100.0
Two-wheelers	100.0	100.0	100.0	100.0	24.9	100.0	100.0	100.0	84.0	76.6	69.9	100.0	69.0	57.4	100.0	100.0
Buses	100.0	100.0	100.0	100.0	22.3	100.0	100.0	100.0	81.6	73.8	64.2	51.2	69.2	61.6	46.4	68.5
Medium-duty trucks	100.0	100.0	100.0	100.0	24.4	100.0	100.0	100.0	80.8	72.1	62.8	49.8	72.5	65.9	43.7	63.3
Heavy-duty trucks	100.0	100.0	100.0	100.0	25.5	100.0	100.0	100.0	82.3	74.1	64.8	53.8	67.1	54.8	47.2	63.3
**Total**	**100.0**	**100.0**	**100.0**	**100.0**	**32.5**	**100.0**	**100.0**	**100.0**	**80.7**	**72.8**	**68.3**	**63.2**	**72.1**	**65.5**	**79.2**	**89.2**

BaU scenario with a 30-year lag																
Light-duty vehicles	100.0	100.0	100.0	100.0	178.5	176.7	100.0	100.0	123.6	136.3	144.6	115.3	109.9	113.2	116.7	100.0
Two-wheelers	100.0	100.0	100.0	100.0	205.3	286.0	100.0	100.0	112.4	119.6	127.2	130.5	125.2	140.2	156.7	100.0
Buses	100.0	100.0	100.0	100.0	229.9	300.4	100.0	100.0	115.6	124.7	137.6	148.4	131.4	147.7	157.3	204.0
Medium-duty trucks	100.0	100.0	100.0	100.0	221.2	281.0	100.0	100.0	116.3	126.3	139.0	149.6	127.6	140.5	149.3	203.9
Heavy-duty trucks	100.0	100.0	100.0	100.0	231.7	265.8	100.0	100.0	115.0	124.5	136.9	146.1	132.9	154.6	176.3	192.8
**Total**	**100.0**	**100.0**	**100.0**	**100.0**	**209.3**	**226.6**	**100.0**	**100.0**	**116.6**	**126.4**	**138.4**	**140.1**	**126.1**	**137.5**	**143.8**	**128.9**

CO_2_ mitigation scenario																
Light-duty vehicles	98.9	92.6	88.8	79.9	98.3	92.6	88.8	80.0	98.1	92.9	87.7	78.3	98.6	92.4	87.4	78.1
Two-wheelers	100.0	100.0	99.9	99.4	100.0	100.0	99.9	94.0	100.0	100.0	100.0	101.0	100.0	100.0	100.0	93.6
Buses	99.3	99.3	98.0	92.6	100.1	99.3	98.0	92.0	100.6	99.3	97.4	101.5	101.8	98.5	95.4	94.0
Medium-duty trucks	99.1	98.0	94.2	77.9	99.5	98.0	94.2	78.0	98.7	97.0	93.5	75.5	98.7	97.6	92.5	68.3
Heavy-duty trucks	99.9	99.8	99.6	84.6	99.9	99.8	99.6	84.6	100.0	100.0	100.0	96.5	99.9	99.9	99.9	93.9
**Total**	**99.3**	**96.0**	**93.4**	**81.7**	**99.3**	**95.9**	**93.3**	**81.6**	**99.5**	**98.2**	**96.2**	**87.8**	**99.7**	**97.6**	**93.7**	**80.4**

CO_2_ mitigation scenario with optimistic cost assumptions																
Light-duty vehicles	79.9	58.0	55.3	46.2	80.6	58.1	55.4	46.4	78.2	53.1	54.0	53.9	75.7	52.3	54.4	53.6
Two-wheelers	100.0	100.0	99.7	100.0	100.0	100.0	99.6	100.0	100.0	100.0	100.0	100.0	100.0	100.0	99.7	100.0
Buses	94.3	93.3	94.0	92.2	93.8	93.5	86.8	70.1	88.1	81.9	83.5	74.6	87.2	83.3	81.7	67.5
Medium-duty trucks	92.3	73.9	49.1	28.2	92.4	73.9	49.2	28.3	83.5	56.3	42.1	36.0	83.5	54.8	38.3	33.7
Heavy-duty trucks	100.1	100.1	99.9	97.9	100.1	100.1	99.9	97.9	100.0	100.0	100.0	99.7	100.1	100.0	99.9	99.5
**Total**	**88.5**	**74.8**	**69.2**	**59.4**	**91.1**	**74.4**	**68.4**	**57.5**	**91.7**	**81.4**	**77.5**	**73.2**	**92.6**	**80.7**	**72.8**	**60.2**

1 This table shows tank-to-wheel CO_2_ emissions. The carbon emission factor for biofuels has been set at zero assuming that biomass is produced in a sustainable way so that they can be regarded as CO_2_ neutral.

**Table A1:** Estimated global fossil energy sources 1.

	Reserves (EJ)	Resources (EJ)	Resource base (EJ)
Coal	37,974	104,377	142,351
Oil			
Conventional 2	6783	12,435	19,218
Unconventional	1926	14,444	16,370
Natural gas			
Conventional 3	5401	13,440	18,841
Unconventional 4	5778	10,802	16,580

1 Denotes global fossil-energy resource bases available for the period to 2100. Resource base is the sum of reserves and resources;

2 Includes natural gas liquids and the potential for enhanced recovery of conventional reserves and resources;

3 Includes the potential for enhanced recovery of conventional reserves and resources;

4 Includes the potential for enhanced coalbed methane recovery.

**Table A2:** Global supply potential of bioenergy in 2000 and 2050 (EJ/year) 1,2.

	2000	2050
Energy crops	20.5	110.1
Modern fuelwood	147.5	122.9
Logging and sawmill waste	9.2	17.3
Black liquor	2.5	4.0
Scrap paper	1.1	0.9
Scrap lumber	5.7	10.9
Grain residues	8.3	17.8
Sugarcane residues	3.7	6.1
Food waste	3.8	6.1
Human excrement	1.3	2.0
Animal manure	3.1	4.8
Waste grease and oil	1.0	1.0

Total	207.6	303.8

1 This estimate was made assuming that all the available excess cropland is allocated to producing energy crops, which are defined as fast-growing trees such as hybrid poplars and willows in the model;

2 The estimates of the availability of various biomass resources and excess cropland (whose evolution is reflected in the values for energy crops in this table) are those that can be used for energy purposes without conflicting with other biomass uses such as the production of food, paper, lumber, and traditional fuelwood. They were estimated assuming that biomass is produced in a sustainable way so that biomass-derived energy carriers can be regarded as CO_2_ neutral.

**Table A3:** Assumptions on CO_2_ emissions from electricity generation 1.

Electricity generation technologies	CO_2_ emissions 2 (t-C/MWh)
Coal-fired steam cycle	0.228–0.186
Coal IGCC	0.220–0.169
Coal-fired IGCC-SOFCs 3	0.166–0.155

Oil-fired steam cycle	0.164–0.138

Natural gas-fired steam cycle/NGCC	0.117–0.085
NGCC-SOFCs 3	0.084–0.079

Light water reactors	0
Fast breeder reactors 4	0

Biomass-fired steam cycle	
using wood chips	0.434–0.293 5
using wood pellets	0.365–0.293 5
using grain residues	0.393–0.266 5
using sugarcane residues (a uniform mixture of bagasse and trash)	0.334–0.226 5
using black liquor	0.660–0.619 5
using municipal wastes	0.475–0.322 5
Biomass IGCC 6	
using wood chips	0.277–0.222 5
using wood pellets	0.262–0.212 5
using sugarcane residues (a uniform mixture of bagasse and trash)	0.218–0.180 5
using black liquor	0.327–0.280 5
Biogas CHP using a gas engine	0.157–0.138 5

Hydrogen-fired power generation using a gas turbine	0

Methanol-fired power generation using a gas turbine	0.151–0.113

DME-fired power generation using a gas turbine	0.134–0.106

CHP by stationary fuel cells	
Hydrogen-fueled PEMFCs used for residential/commercial applications	0
Natural gas-fueled PEMFCs used for residential/commercial applications	0.169–0.138
Hydrogen-fueled SOFCs used for residential/commercial applications 6	0
Natural gas-fueled SOFCs used for residential/commercial applications 6	0.135–0.100
Hydrogen-fueled SOFCs used for industrial applications 6	0
Natural gas-fueled SOFCs/MCFCs used for industrial applications 6	0.117–0.087

Hydropower	0
Geothermal power	0
Wind power	0
Solar power	0

1 IGCC = integrated gasification combined cycle; NGCC = natural gas combined cycle; SOFC = solid oxide fuel cell; PEMFC = proton exchange membrane fuel cell; MCFC = molten carbonate fuel cell;

2 These ranges denote the assumed evolution of the parameter values over the time horizon;

3 Assumed to be available from 2030;

4 Assumed to be available from 2050;

5 It is assumed that CO_2_ emissions created from biomass burning are offset by biomass growth;

6 Assumed to be available from 2020.

**Table A4:** Assumptions on CO_2_ emissions from biofuels production.

Biofuels production technologies	CO_2_ emissions (t-C/TJ-fuel)	
	2000	2050
Bioethanol production		
from high-quality woody biomass 1	0 2	0 2
from wood pellets 1	0 2	0 2
from corn	7.66 2	7.66 2
from wheat	12.42 2	12.42 2
from sugarcane	0 2	0 2
from sugarbeet	8.55 2	8.55 2
from cellulosic waste biomass 1	0 2	0 2

Biodiesel production	5.07 2	5.07 2

Biogas production	0 2	0 2

Hydrogen production 1		
from high-quality woody biomass	45.72 + 22.94α 3,4	40.33 + 20.23α 3,4
from black liquor	54.84 + 0.624α 3,4	48.34 + 0.550α 3,4

Methanol production 1		
from high-quality woody biomass	32.02 + 11.18α 3,4	26.08 + 9.87α 3,4
from black liquor	34.65 3	28.38 3

DME production 1		
from high-quality woody biomass	34.78 + 14.37α 3,4	28.58 + 12.69α 3,4
from black liquor	34.17 3	28.02 3

Raw FT liquids production 1		
from high-quality woody biomass	40.24 + 9.19α 3,4	33.19 + 8.11α 3,4
from black liquor	40.73 3	33.59 3

1 Assumed to be available from 2020;

2 Denotes net CO_2_ emissions;

3 Same as footnote 5 in Table A3;

4 α denotes the average CO_2_ emission factor of the electric power grid in a model region (in t-C/MWh).

## Appendices

### A1. Detailed Description of the Assumptions Underlying the REDGEM70 Model

#### A1.1. Assumptions on the Availability of Primary Energy Resources

Table A1 shows the assumptions on the availability of fossil energy sources, while Table A2 shows the assumptions on the availability of biomass resources.

#### A1.2. Assumptions on the Maximum Allowable Market Penetration Rate of New Technologies

Figure A1 shows the assumed maximum allowable market penetration of new transport technologies available for light-duty vehicles that are not existent until now. The maximum allowable market penetration rate of new transport technologies available for light-duty vehicles is represented by the following constraint:

$$\begin{array}{l} \sum_{s}2*LF(\text{LDV},i,t-1)*ADT(\text{LDV},i,t-1)*vin(\text{LDV},s,t-1)*V(\text{LDV},v,i,s)+0.1*Ract(\text{LDV},i,t)+0.000001\\ \geq \sum_{s}LF(\text{LDV},i,t)*ADT(\text{LDV},i,t)*vin(\text{LDV},s,t)*V(\text{LDV},v,i,s) \end{array}$$

For the definition of constants and variables, see Section 2.2. The maximum allowable market penetration of new transport technologies available for light-duty vehicles that are not existent until now can be derived from this constraint, as shown in Figure A1. The maximum allowable market penetration shown in Figure A1 is consistent with that in the aggressive scenario used in [43]. The same constraint is imposed on the maximum allowable market penetration rate of new transport technologies available for the other transport modes.

#### A1.3. Assumptions on CO_2_ Emissions from Electricity Generation and Biofuels Production

Table A3 shows the assumptions on CO_2_ emissions from electricity generation, while Table A4 shows the assumptions on those from biofuels production.

#### A1.4. Assumptions on the Average Annual Distance Traveled per Vehicle

Figure A2(a),(b) shows the assumed average annual distance traveled per vehicle for light-duty vehicles and buses, respectively, by world region. The average annual mileage for two-wheelers and trucks was set based on [1], which is assumed to remain unchanged over the time horizon. For twowheelers, the average annual distance traveled per vehicle was set at 5000 km/year for North America, 7500 km/year for the other developed regions, and 10,000 km/year for the reforming and developing regions. For medium-duty trucks, it was set at 30,000 km/year for the developed regions and 20,000 km/year for the reforming and developing regions. For heavy-duty trucks, it was set at 60,000 km/year for all world regions.

### A2. Simulation Results not Included in the Main Text

Figure A3 shows the shadow prices of petroleum gasoline and diesel fuel for the three scenarios that are calculated as weighted global averages derived from the results for 70 world regions, while Figure A4 shows the cost-optimal mix of transport technologies available for light-duty vehicles for the three scenarios.

It has been confirmed that if the cost of gasoline plug-in hybrid electric light-duty vehicles is reduced by 16.8% (15.0%) from that assumed in this study, then they hold a share of 10% in the global light-duty vehicle fleet in 2050 in the BaU scenario (CO_2_ mitigation scenario).

Sensitivity analysis was performed by additionally imposing prices on using petroleum gasoline and diesel fuel which are the same as or twice their shadow prices derived from the model. In the scenarios with a 100% price increase, the REDGEM70 model was run by imposing additional prices on using petroleum gasoline and diesel fuel which are the same as their shadow prices, while it was run by imposing additional prices on using them which are twice their shadow prices in the scenarios with a 200% price increase. The results of this sensitivity analysis are shown in Figure A5 and Figure A6. Figure A5 shows the cost-optimal mix of road transport fuels for the six scenarios, while Figure A6 shows the global emissions of CO_2_, SO_2_, NO_x_, and PM from road vehicles for the six scenarios.
